# Towards an Evolutionary Understanding of Questing Behaviour in the Tick *Ixodes ricinus*

**DOI:** 10.1371/journal.pone.0110028

**Published:** 2014-10-15

**Authors:** Joseph L. Tomkins, Jennifer Aungier, Wade Hazel, Lucy Gilbert

**Affiliations:** 1 Centre for Evolutionary Biology, School of Animal Biology, University of Western Australia, Crawley, Western Australia, Australia; 2 Institute of Biological and Environmental Sciences, Zoology Building, University of Aberdeen, Aberdeen, United Kingdom; 3 Department of Biology, Depauw University, Greencastle, Indiana, United States of America; 4 James Hutton Institute, Craigiebuckler, Aberdeen, United Kingdom; University of Tours, France

## Abstract

The tick *Ixodes ricinus* finds its hosts by climbing vegetation and adopting a sit-and-wait tactic. This “questing” behaviour is known to be temperature-dependent, such that questing increases with temperature up to a point where the vapor pressure deficit (drying effect) forces ticks down to rehydrate in the soil or mat layer. Little if any attention has been paid to understanding the questing of ticks from an evolutionary perspective. Here we ask whether populations from colder climatic conditions respond differently in terms of the threshold temperature for questing and the rate of response to a fixed temperature. We find significant variation between populations in the temperature sensitivity of questing, with populations from cooler climates starting questing at lower temperatures than populations from warmer temperatures. Cool climate populations also quest sooner when the temperature is held constant. These patterns are consistent with local adaptation to temperature either through direct selection or acclimation and challenge the use of fixed thresholds for questing in modeling the spread of tick populations. Our results also show how both time and temperature play a role in questing, but we are unable to explain the relationship in terms of degree-time used to model Arthropod development. We find that questing in response to temperature fits well with a quantitative genetic model of the conditional strategy, which reveals how selection on questing may operate and hence may be of value in understanding the evolutionary ecology of questing.

## Introduction

While understanding the ecology of pathogen vectors is vital to increasing our ability to forecast changes in disease risk, changes in range and prevalence in response to changes in climate will also involve evolution [1]. Vector-borne diseases are particularly likely to be impacted by climate change, since the Arthropod vectors (such as ticks, flies and mosquitos) are strongly affected by temperature and climatic conditions [2], [3]. The effects of changing climate on the distribution and prevalence of disease is a major concern both for the health of human populations and the health and persistence of populations of domestic and wild animals and plants [4]–[8].

The sheep tick, *Ixodes ricinus* (Arthropoda: Acari) is the principal vector of the tick-borne encephalitis complex of viruses (including louping-ill virus), *Rickettsia, Babesia*, *Anaplasma* species and the *Borrelia burgdorferi* complex of bacteria, the causative agent of Lyme borreliosis. The expansion of *I. ricinus*' European range in recent decades to higher latitudes and altitudes, concomitant with increases in its population density [9], [10], and increases in reported cases of tick-borne diseases in some areas [11], have been partly attributed to changes in climate. There is speculation and some concern that climate change already has influenced, and will continue to influence, *I. ricinus* and the epidemiology of the pathogens it transmits [2], [3], [12].

‘Questing’ in ticks involves the tick leaving the ground microhabitat of moss, leaf-litter and detritus and climbing up vegetation, where it adopts a sit-and-wait tactic for finding a host. Temperature and relative humidity requirements for *I. ricinus* questing, development, and survival are thought to be the principal factors limiting the geographic range of the species [10], [13], [14]. Since questing is the behaviour that puts humans and livestock at risk of parasitization and pathogen infection, understanding the ecology and evolution of questing is key to understanding the changes in risk of tick borne diseases with a changing climate [15].

The environmental conditions that influence questing behaviour have been widely explored, and temperature is considered the most important factor influencing the initiation of *I. ricinus* questing [16]–[19]. At low temperatures, the assumption is that ticks cannot function metabolically at a sufficient level to quest [20], and this sets the lower limit to questing [20]. In contrast, at higher temperatures and lower relative humidities the resulting high vapor pressure deficit (the ‘drying power' of the air; [17] leads to the termination of questing activity and ticks descend to the moist litter-layer to actively re-absorb water [18]. The suggestion is that these physiological thresholds constrain the distribution of tick populations. While the idea of fixed temperature thresholds applying across populations may be a convenient assumption from the point of view of predicting the distribution of ticks [13], [14], [21], [22], it may lack realism [23], [24]. Evidence from threshold traits in invertebrates, including Acari, show that thresholds can evolve over timescales of a relatively few generations [25]. Indeed we have recently shown how geographically separate populations of *I. ricinus* show clinal variation in the response of questing to temperature, suggesting that physiological thresholds are not fixed [26].

**Figure 1:**
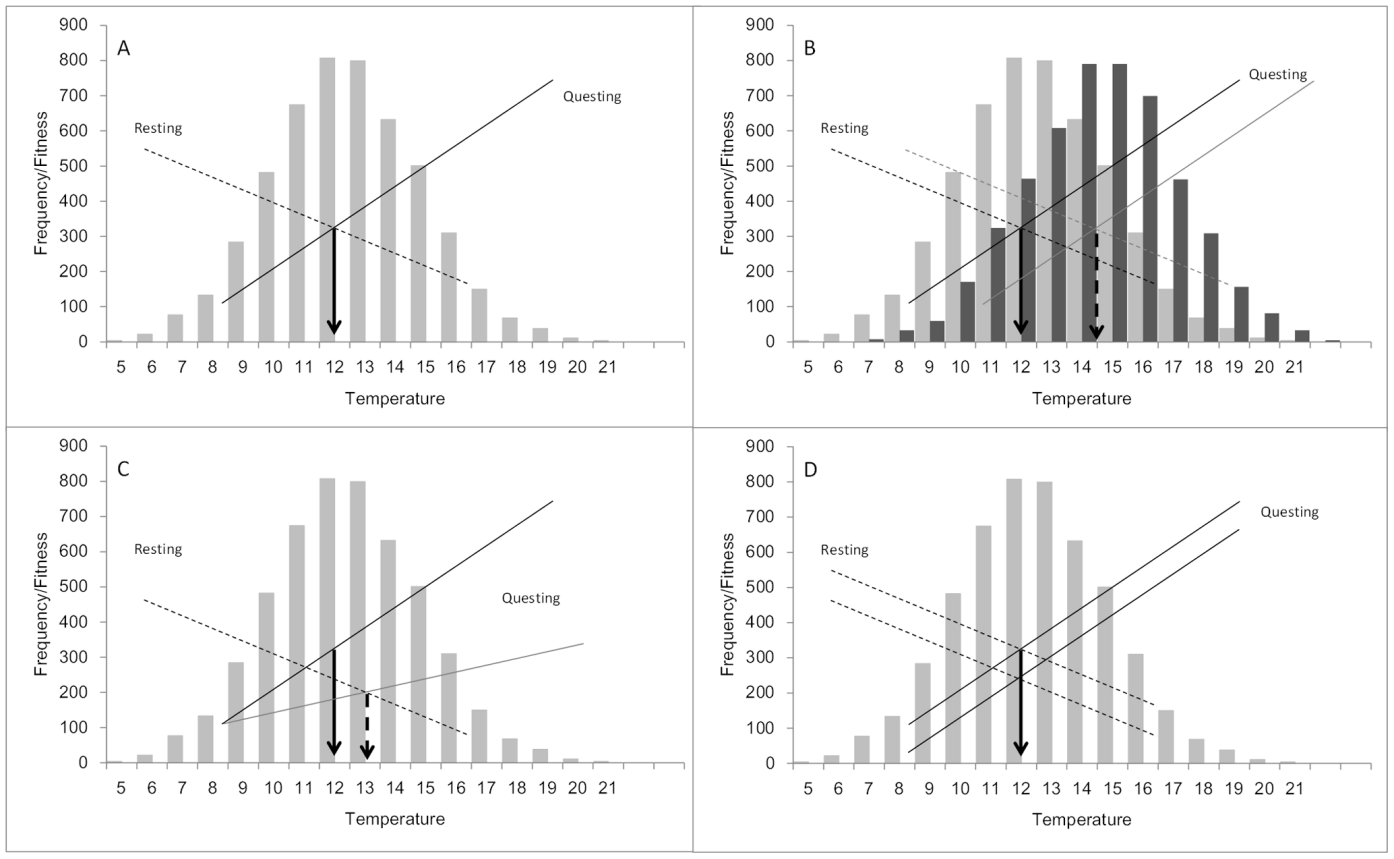
Environmental threshold model for tick questing behaviour. **a**) Theoretical expressions of the fitness functions (straight lines) for questing and resting ticks and the frequency distributions of temperatures (bars) in a tick population. We assume that resting ticks face declining fitness from missing opportunities to find a host as temperature increases (solid line). As temperature declines, we assume that questing ticks face costs such as an increased probability of wasting resources searching for hosts when they are too cold to move rapidly enough to grasp them, as well as increased predation risk when they can only move slowly (dashed line). The combination of these effects results in selection for switch points distributed around the temperature at which fitness from questing first begins to exceed the fitness from resting (arrow). **b**) A difference or change in the distribution of temperatures (pale bars vs dark bars) should be accompanied by a parallel shift in the fitness functions (black vs grey lines) and therefore a proportional change in the mean of the switch point distribution (arrows). **c**) Temperature-dependent effects on the fitness of one of the phenotypic states (e.g. questing), will alter the slope of the fitness function (from solid black line to solid grey line) this will alter the switch point favoured by selection and result in an evolutionary shift in the switch point distribution to the new average switch point temperature (from solid arrow to dashed arrow). **d**) Temperature independent effects e.g. a reduction in host numbers, will affect the fitness of both phenotypes equally (dashed lines) and will not alter the switch point temperature (arrow) that is favoured by selection.

The questing behaviour of ticks can be viewed as a switch between two dichotomous states, questing or not questing, with environmental conditions such as temperature affecting when ticks switch between the two (e.g. Figure 1). When environmental cues affect phenotypic switching (e.g. from not questing to questing), the evolution of the sensitivity of the switch to the cue can be modeled as an environmentally-cued threshold trait [27]–[29] using the environmental threshold model [27]. The model describes how the mean switch point of a population evolves by assuming individuals vary genetically in the cue-strength that is needed to trigger the switch from one phenotype to another [27], [29]. Three key parameters in the model determine the evolutionarily stable switch point: (i) the variance in the switch point distribution (i.e. within a population of ticks, the genetic variation in sensitivity of questing to temperature), (ii) the fitness returns from questing and not questing, and (iii) the distribution of the cue (i.e. the frequencies of particular temperatures that the population experiences). Hence, the environmental threshold model can provide a framework for understanding how natural selection leads to local adaptation in questing [e.g. 25,30,31]. Figure 1, illustrates how the environmental threshold model can be applied to the question of how questing in response to temperature might vary with climate or climate change [26].

Under the model, the fitness of questing increases with temperature, while that of not questing decreases with temperature (Figure 1a). The model predicts that the distribution of questing switch points will track the intersection of the fitness functions (Figure 1b), although a one-to-one relationship between mean switch point and the intersection of the fitness functions is unlikely [29]. The temperature dependence of the initiation of questing (and its termination) allows individuals to adaptively allocate their time to questing and water reabsorption, because both activities are necessary for finding a host. In warm regions, temperature constraints on initiation of questing are less severe than in cooler regions (although termination may be under stronger selection in warmer regions). In cooler regions individuals that can initiate questing at a lower temperature will have a fitness advantage because they can more adaptively allocate the amount of time spent questing versus water reabsorption. Therefore, for populations in cooler regions we expect the fitness functions of questing and not questing to intersect at a lower temperature relative to populations in warmer regions (Figure 1b). If switch variation is heritable, then the time of initiation of questing is expected to reflect this difference (Figure 1b). The slopes of the fitness functions (in addition to their intersection) could also be influenced by temperature-dependent effects, such as the temperature–dependent activity of hosts (Figure 1c). For example, if different host communities have different temperature-dependent patterns, any changes in climate that alter host communities may have fine scale effects on questing. However, if adaptive temperature-dependent allocation of time to questing is the primary factor driving selection on the initiation of questing, the model predicts that questing initiation and climate will covary (Figure 1b). Effects on fitness functions that are not temperature-dependent, e.g. changes in host abundance, should affect only the elevation (rather than the slope) of the fitness function(s) and will not affect the average switch point (Figure 1d).

The environmental threshold model assumes that in a population, switch point variation can be treated as a typical quantitative trait. Therefore, assuming that temperature itself is the cue triggering the initiation of questing, the expectation is that for a given population, as temperature increases, the fraction of individuals questing will smoothly increase to 100%, with the temperature at which 50% questing occurs estimating the mean switch point of the population [29]. The more variation there is in the switch points of a population, the slower the increase in the fraction questing as temperature increases. Therefore, if populations differ in either mean or variance in switch points, their cumulative response of questing to temperature will differ.

Although questing in ticks is strongly influenced by temperature, arthropod development, in general, is dependent on the integration of temperature with time; once a critical temperature has been reached, the organism accumulates ‘metabolic time’ as a function of the time spent above the threshold for metabolic activity ‘*T_metab_*‘ and of how much the current temperature, ‘*T_current_*’ exceeds *T_metab_* [32, and methods for details]. Such an integration of time and temperature (e.g. degree days) is widely used to understand the phenology of insect pests [33], [34], and in ticks is supported by developmental experiments [15], [35], and is used in predicting tick distributions [14], [22]. Field surveys for ticks in Scotland [16], Switzerland [17] and northern Italy [36] show that ticks become active in large numbers only after winter diapause when the weekly average of the maximum daily air temperature is above 7 or 8°C [15]. Hence tick activity and development may be dependent not solely on temperature, but also on time- specifically on the amount of time that the tick has spent above a metabolically significant temperature. The extent to which time and temperature are integrated to determine the initiation of questing can be addressed by examining the cumulative questing responses of ticks when plotted against metabolic time in ticks exposed to hourly increasing temperatures versus a constant temperature. If metabolic time switch points are influencing questing, then the response curves in the treatments should be similar.

Here we demonstrate the utility of controlled laboratory experiments in understanding patterns of variation within and between geographic tick populations. Our expectation is that temperature-dependent questing is exposed to selection and that ticks from cooler climates should initiate questing at lower temperatures than ticks from warmer climates; something we recently verified [29]. Here we show how distributions of questing activity within populations can be quantified in a laboratory setting and interpreted in the light of the environmental threshold model, which we advocate as a tool for understanding local adaptation in the initiation of questing [29]. We also investigate the hypothesis that time, as well as the temperature, above a metabolically significant threshold temperature combine to explain variation in questing, with the prediction that patterns of questing in constant temperatures and those in increasing temperatures can be reconciled by combining time and temperature with the ‘degree time’ formula used to model Arthropod development. The overarching aim is to build an evolutionary genetic framework for understanding temporal and spatial variation in questing.

## Materials and Methods

### Ethics statement

No endangered species and no vertebrates or other species requiring ethics approval were collected or disturbed during this study.

### Tick collection - locations and techniques

*I. ricinus* were collected from five sites with different thermal climates:- North East Scotland, Northern Wales, Southern England and both a high and a low altitude site (50 km apart) in central France [Table 1: see also [26]]. Permissions to collect ticks were obtained from The James Hutton Institute (North East Scotland), The New Forest National Park Authority, Lymington Town Hall, Avenue Road, Lymington, SO41 9ZG, UK (Southern England). Ticks were collected on open access land in North Wales and France.

**Table 1:** Location and climate details of the five geographic locations from which ticks were collected (see methods for estimation of metabolically significant maximum temperature).

Region	Latitude & longitude	Alt (m)	Estimated yearly mean max. temp.	Estimated metabolically sig. max. temp.
NE Scotland	56° 54′N, −2° 31′E	300	9.9°C	11.6°C
N Wales	53° 5′N, −3° 15′E	230	12.4°C	14.4°C
S England	50° 51′N, −1° 38′E	40	13.8°C	13.8°C
C France High	45°40′N, 03°46′E	1120	11.3°C	13.9°C
C France Low	45°47′N, 03°27′E	380	16.0°C	17.7°C

“Estimated yearly mean max. temp.” refers to the annual averages of the maximum temperatures recorded for each month for the period 1971–2000.

To control for inter-stadial differences in temperature thresholds [2], [20] only nymphs were used in our experiments. All nymphs were collected within the last 2 weeks of May 2012. Nymphs were collected from all sites by blanket dragging [37]. Field conditions during dragging ranged from 17–22°C and 60–70% relative humidity (recorded using Tinytag digital data loggers). Nymphs were stored in plastic boxes (5×10 cm), containing damp tissue to maintain high humidity, and kept at 4–6°C prior to the experiments.

In order to minimize stimulation of questing in response to host stimuli (e.g. CO_2_, vibrations, and shadows), incubators were located in a quiet laboratory and their internal glass door was kept closed during observations. Observers moved slowly and quietly during counts. Two *Tinytag* digital data loggers were used to calibrate each incubator and to subsequently log the temperature and relative humidity inside the incubators every 5 minutes. These data showed humidity was maintained at 90–100% and the incubators took ∼10 minutes to stabilize from one temperature to the next.

Questing tubes were made from glass cylinders (0.6 cm diameter, 14 cm length), the base of each filled with 2 cm of plaster of Paris. The cylinders were stood in cold water for 2 minutes in order to moisten the plaster of Paris prior to experiments, and then stood briefly on paper towel to remove excess water and reduce condensation in the tube. A single tick was placed into each tube and the top plugged with a damp cotton wool bung. The damp plaster and cotton wool ensured a constant high humidity was maintained inside the glass tubes. Within a portable incubator (Memmert IPP 200), 115 glass tubes were arranged near vertically (±85°) in two rows one, above the other, against a white background (to maximize the visibility of ticks within the tubes). To minimize any variation related to position within the incubator glass tubes were arranged in rows with population of origin alternating along each row. Due to limited space inside each incubator we repeated the experiments using the same incubators and tubes but with different ticks and included ‘block’ as a factor in our analysis. To avoid the bio-security issue of transporting live specimens across borders, the high and low altitude French populations were tested in France two weeks prior to the UK populations. -. The three UK populations were tested in three blocks. In both the UK and France 115 ticks were tested from each population.

Following set-up, a ‘summer-time’ light regime was initiated (7 hrs dark: 17 hrs light, to allow all observations to be made under daylight conditions). Glass tubes were left overnight (8 hours) at 1°C, to initially discourage questing activity. At ‘lights on’ the initial vertical position of each tick within its tube was recorded (0–12 cm above plaster base of tube). The temperature was increased to 5°C for an hour, and vertical positions re-recorded. The temperature was then raised by 1°C each hour and the vertical position of each tick re-recorded hourly (prior to the next increase in temperature), for a further 16 hours, up to 21°C. Hence the temperature and time at which each tick first moved >0.5 cm was recorded, and this point in time/temperature was taken as that tick's questing temperature or questing time. This hourly temperature increase was chosen as it allowed us to monitor the activity of individuals over a wide range of temperatures, similar to the range experienced within a single day by *I. ricinus* populations in their natural environments.

### Degree-time

In order to examine the hypothesis that time at a particular temperature as well as the temperature itself, is important, two constant temperature trials were conducted. The above experimental set-up and observations were repeated exactly, however, the temperature was held constant throughout the experiment, once at a constant 9°C and again at a constant 15°C for 16 hours using 38 ticks from each of the three UK populations.

To estimate degree-time we first needed to estimate the temperature below which no activity is possible *T_metab_* for each population. For each population, we used the mean and standard deviation of the observed distribution of temperatures at which questing was initiated, to generate a cumulative probability distribution of questing initiation. We used this distribution to estimate the temperature at which 1% of the population had started to quest. This provided our estimate of *T_metab_*. Our estimates of *T_metab_* were 5.7°C for Scotland, 7.9°C for Wales, 7.0°C for England, 9.3°C for France low altitude and 6.9 for France high altitude.

We estimated the number of degree hours required to initiate questing in both the constant temperature treatment and increasing temperature treatment as
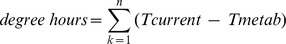
where *T_current_* was the temperature in the incubator during a given hour, n =  number of hours at which *T_current_* was greater than *T_metab_* and k =  the first hour at which *T_current_* exceeded *T_metab_*. The difference between the constant and increasing temperature treatments is that the term in parentheses is constant in the constant temperature treatment but varies in the increasing temperature treatment. For example, where the temperature is 15°C and *T_metab_* is 10°C, at a constant temperature for five hours would result in 25 degree hours ((15−10)*5), whereas with temperature increasing hourly up to 15°C for five hours, the result is 15 degree hours ((11−10)+(12−10)+(13−10)+(14−10)+(15−10)).

### Weather station data

To estimate the local thermal climatic conditions typically experienced by the five tick populations in their natural environments, 1971–2000 long term average climate data was obtained from the UK Met Office [38] and from Meteo France, via the World Weather Information Service [[39]; Table 1]. Data came from the nearest weather station to each location: Craibstone, 102 meters above sea level (masl), 35 km NNE of the NE Scotland collection site; Shawbury, 72masl, 50 km SE of the N Wales collection site; Everton, 16masl, 13 km S of S. England collection site; Clermont Ferrand, 331masl, 50 km E of France High altitude collection site, and 25 km E of France Low altitude collection site. The mean monthly maximum temperatures recorded at these stations were adjusted to account for differences in altitude; subtracting 6.4°C for every 1000 m elevation gain. We used maximum temperatures because average temperatures (max+min/2) would have included temperatures that were below T*_metab_* and therefore irrelevant for tick activity. In addition to the average monthly maximum temperatures we also report the estimated “metabolically significant” maximum temperature. This was estimated by calculating the average temperature using only those months where the average maximum temperatures were above our estimate of T*_metab_* (Table 1).

### Statistical analyses

To test whether the temperature of questing initiation varied significantly between populations the temperature at which each tick first climbed ≥1 cm above its starting position (i.e. position after 8 hrs at 1°C) was recorded. Ticks that did not move were excluded from this analysis [40]. Graphical exploration revealed no outliers and a normal distribution of the response variable (temperature at initiation of questing). A mixed effect model was fitted, using the MIXED procedure in SAS version 9.1.3 [41]. Model assumptions of homogeneity and normality of residuals were met. The mixed model was run to analyse the variation in temperature at initiation of questing activity, with ‘population’ as a fixed effect, and block as a random effect (accounting for the five runs of the experiment required to test 115 ticks from each populations).

To analyse the patterns of questing with temperature we used the data for all ticks whether they moved or not. We used self-starting non-linear regression models in R [42] to describe the cumulative normal distribution of questing in relation to hourly increases in temperature (i.e. the cumulative proportion of questing ticks in the population). We tested the family of self-starting nonlinear models (available in the *Selfstart* package, [43]) and the *initrichards* function in the *grofit* package [44] for the initial values of the Richards equation (below) and compared them based on their AIC values (lower AIC indicates better fit model). The four parameters of the Richards equation are:
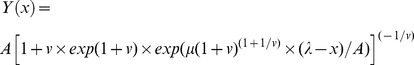
where: *A* is the asymptote of the curve, ν is a shape parameter describing how close the maximum slope is to the asymptote, µ is the maximum slope of the curve, and λ is an estimate the value of × (i.e. temperature) at the end of the lag phase.

To find the best fitting three or four-parameter model, the parameters were initially allowed to vary between populations such that each population could have different parameter values from the others. The AIC of this model was then compared to a model in which one of the parameters was ‘fixed’ (not allowed to vary between populations), and the AIC again computed. By working through all of the parameters and all of the combinations of parameters the best fitting model (lowest AIC) was determined.

## Results

### Population variation in average switch point

**Figure 2:**
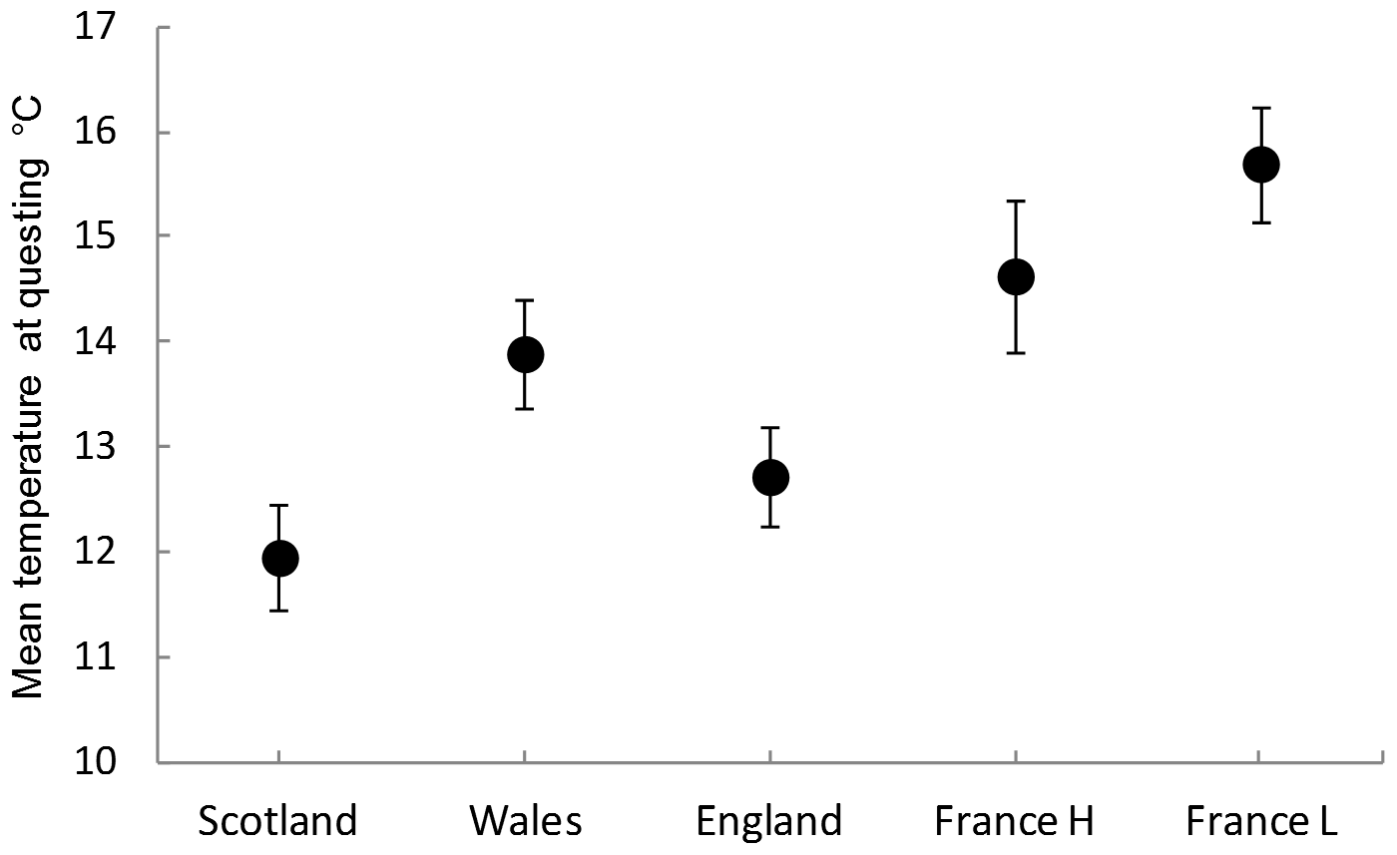
Mean ±95% CI temperature at which questing was initiated in each tick population during the hourly temperature increase experiments. H and L =  High and Low altitude.

The average initiation temperature of questing, a measure of the mean switch point, varied significantly between populations (F_4, 466_  = 12.81, P<0.0001). As predicted under the hypothesis that questing has evolved to local thermal conditions rather than representing a metabolic constraint, based on annual mean monthly maximum temperatures, populations from the cooler thermal climates had significantly lower mean switch points than those from warmer climates (Ordered Heterogeneity test [45] r_s_P_c_ = 0.599, P<0.04 Figure 2 and Table 2). N. Wales and S. England provide some interesting variation to this pattern; although the thermal climate of N. Wales is slightly (1–2°C) cooler than, and most similar to, that of S. England (according to our estimated mean monthly maximum temperatures), the S. England population was found to have a significantly lower average initiation temperature for questing activity (12.9°C) than the N. Wales population (13.7°C) (Tukey-Kramer test, P = 0.0143; Figure 2, Table 2). However, the climate in the south of England is less variable than in North Wales, whose monthly mean maximum temperature exceeds *T_metab England_* in every month of the year. If only the mean monthly temperatures that exceed *T_metab_* are analysed the Welsh population experiences a warmer climate above *T_metab_* than the English one. Based on average maxima above T*_metab_*, the climate and the average questing temperatures correlate more strongly (Ordered Heterogeneity test; r_s_P_c_ = 0.999; P<0.001), further supporting the hypothesis that cooler climate ticks quest at cooler temperatures. The comparisons above include the low and high altitude French populations, although they were assayed at a different time from the UK populations. A separate analysis of the French populations only revealed that in the low altitude (warmer climate) population the average questing temperature was significantly warmer than that of the high altitude (cooler climate) population (F_1,157_ = 5.267, P = 0.023).

**Table 2:** Results of Tukey-Kramer comparisons of the least square mean temperature at initiation of questing between tick populations.

Population 1	Population 2	t-value	*P*-value
S. England	France High Altitude	−2.95	0.027
S. England	France Low Altitude	−4.65	<0.001
S. England	NE. Scotland	2.04	0.250
S. England	N. Wales	−3.16	0.014
France High Altitude	France Low Altitude	−2.50	0.092
France High Altitude	NE. Scotland	4.13	0.001
France High Altitude	N. Wales	1.12	0.798
France Low Altitude	NE. Scotland	5.85	<0.001
France Low Altitude	N. Wales	2.79	0.043
NE. Scotland	N. Wales	−5.24	<0.001

Significance level *P*<0.05.

### Quantifying the shape of the cumulative response to increasing temperature

The cumulative questing distribution (the increase in the total number of ticks questing with increasing temperature) can be used to estimate parameters of interest when comparing variation in thresholds among populations. We applied the four-parameter Richards equation (see below for details of what the parameters mean) to these data where all parameters were allowed to vary between populations (see methods) (df = 21, AIC = 315.2). This model was significantly superior to the best fitting three parameter nonlinear model (*SSlogis*) despite the extra parameter weighing against it in the calculation of AIC (df = 16, AIC = 319.3). From here, searched for a better fitting model by restricting the combination of parameters that were allowed to vary between populations (see methods). However, again, allowing all parameters to vary yielded the best fit compared to holding any combination constant (the next best fitting model allowed A, µ and λ, but not ν, to vary (df = 17, AIC = 321).

**Figure 3:**
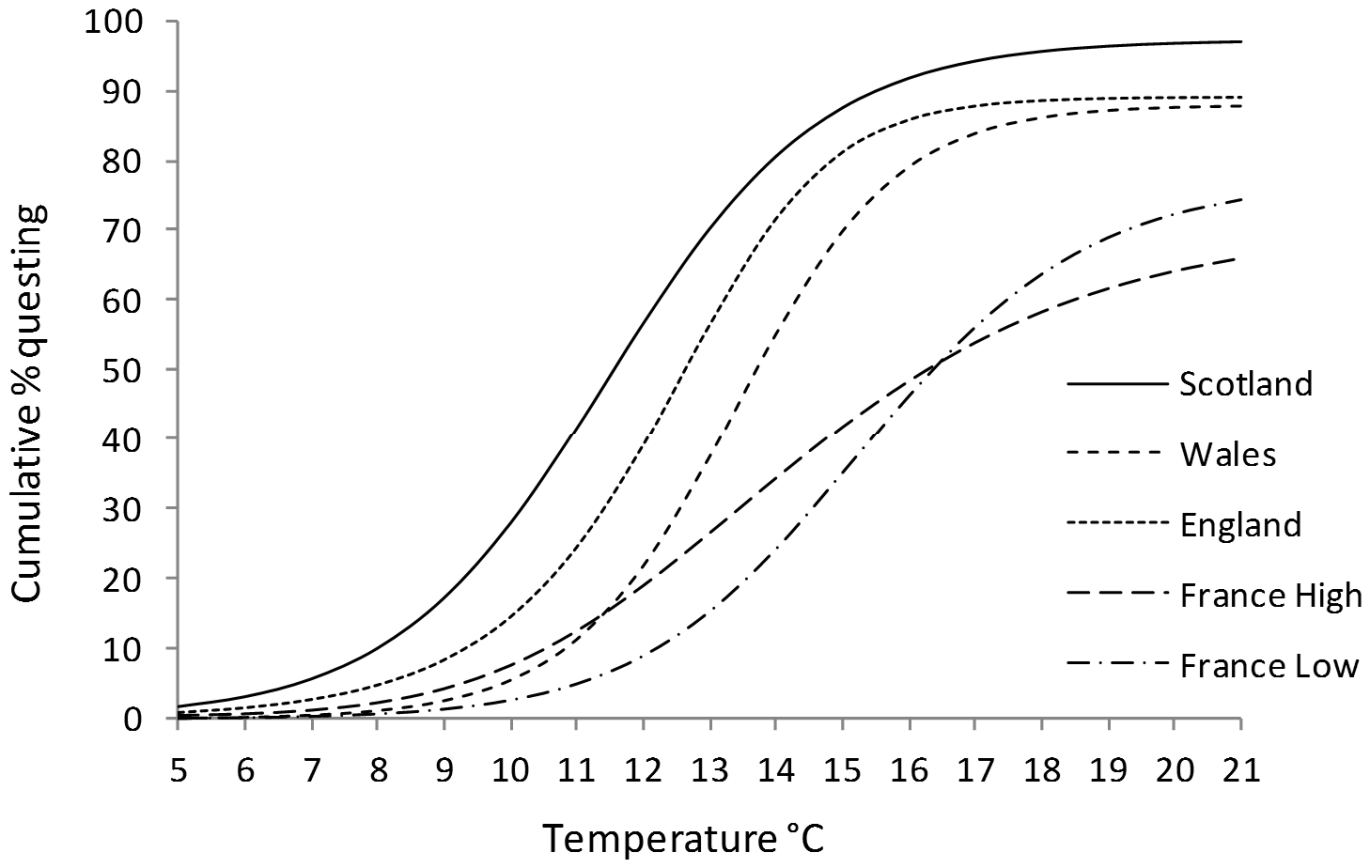
Curves fitted to the predicted values from the Richards equation, for the cumulative % of *I. ricinus* in each population to initiate questing over the course of the hourly temperature increase experiments.

The individual parameters in the Richards equation reflect different descriptors of the cumulative questing distribution. Each population's parameter estimates are reported in Table 3 and resulting estimates of the cumulative questing distributions are shown in Figure 3. A is the asymptote and reflects the point at which temperature increases no longer cause an increase in the proportion of ticks questing. Hence, the populations differ significantly in asymptote (i.e. the maximum response to increasing temperature): ticks from Scotland showed the greatest response and those from the France low altitude site showed the least response to rising temperatures (Table 3).

**Table 3:** Parameter estimates from the Richards equation for the cumulative questing distribution, where λ is the temperature at which questing suddenly starts to increase, µ is the maximum slope of the curve, ν is the is a shape parameter describing how close to the asymptote the maximum slope of the curve is and A is the Asymptote.

Population	λ	L95%CI	U95%CI	µ	L95%CI	U95%CI	ν	L95%CI	U95%CI	A	L95%CI	U95%CI
Scotland	8.26^a^	8.04	8.48	15.14^a^	14.31	15.97	0.98^a^	0.59	1.37	97.14^a^	95.58	98.70
Wales	10.93^b^	10.72	11.14	18.08^b^	16.83	19.33	1.08^a^	0.60	1.57	88.08^b^	86.48	89.68
England	9.80^c^	9.57	10.04	17.67^b^	16.42	18.91	1.7^b^	1.10	2.31	88.97^b^	87.64	90.30
France L	11.92^d^	11.59	12.25	11.42^c^	10.45	12.38	0.82^a,c^	0.25	1.39	77.44^c^	73.26	81.62
France H	9.69^c^	9.29	10.08	7.92^d^	7.25	8.59	0.34^c^	−0.16	0.83	70.50^d^	65.29	75.71

L and H =  Low and High altitude populations. L95%CI and U95%CI  =  lower and upper 95% confidence intervals respectively.

Parameter estimates that share the same superscripts do not differ significantly from one another.

In the Richards equation ν is a shape parameter describing how close the maximum slope is to the asymptote while µ is the maximum slope of the curve. Together ν and µ can be informative about the variance in the switch point distribution – steeper slopes where ν is small are indicative of less switch point variance in a population. The high altitude French population had the lowest values of µ and ν suggesting that this population has more variance in the response to temperature than the others. This is borne-out by examination of the raw questing data, the high altitude French site having a significantly greater standard deviation, than the other sites (one sample t test against the standard deviation of the France high altitude site; t_3_ = 11.3, P<0.001).

λ provides an estimate of the temperature at which the ‘lag phase’ ends and is therefore the temperature above which ticks become increasingly active. λ was lowest in Scotland and highest in lowland France in parallel with the average temperature at the initiation of questing (Figure 2). Our estimate of *T_metab_* correlates strongly with λ as might be expected, but λ is higher than *T_metab_* by 2.7°C±0.08. Wales and England differed in λ in the same manner as they did with the average temperature at initial questing. The English and the high altitude French populations did not differ in λ, reflecting the high variance in λ in the high altitude French population (Table 3).

### Normality and variance of switch point distributions

Under the environmental threshold model, the polygenic basis of the switch point distribution is assumed to give rise to a normal distribution of switch points. The switch point distributions of the ticks from each population were tested for normality for each experimental block by subtracting the mean temperature at questing from each observation to control for variation in means between blocks, and then testing the distribution with a Shapiro-Wilk test (Table 4). Only the Welsh population diverged from normal after correcting for multiple tests. This divergence was caused by leptokurtosis (Table 4).

**Table 4:** Summary statistics for the distributions (and shape of the curve) of “temperature at first questing” (°C) for five populations of *I. ricinus* from Scotland southward to France.

Population	Mean	Sdev.	Shapiro-Wilk	df	P	Skewness	SE	Kurtosis	SE
Scotland	11.95	2.67	0.99	108	0.489	0.04	0.46	0.00	0.26
Wales	13.89	2.57	0.96	101	0.002	−0.29	0.24	1.77	0.48
England	12.72	2.40	0.98	102	0.183	0.09	0.24	0.83	0.47
France H	14.63	3.17	0.97	75	0.103	−0.73	0.55	0.00	0.28
France L	15.70	2.56	0.99	85	0.464	−0.13	0.26	−0.25	0.52

Mean and standard deviation of the initiation of questing (Sdev.) controlling for experimental blocks, Shapiro Wilk test for the normality of the temperature at questing, Skewness and Kurtosis and their respective standard errors (SE).

### Testing the degree time hypothesis

**Figure 4:**
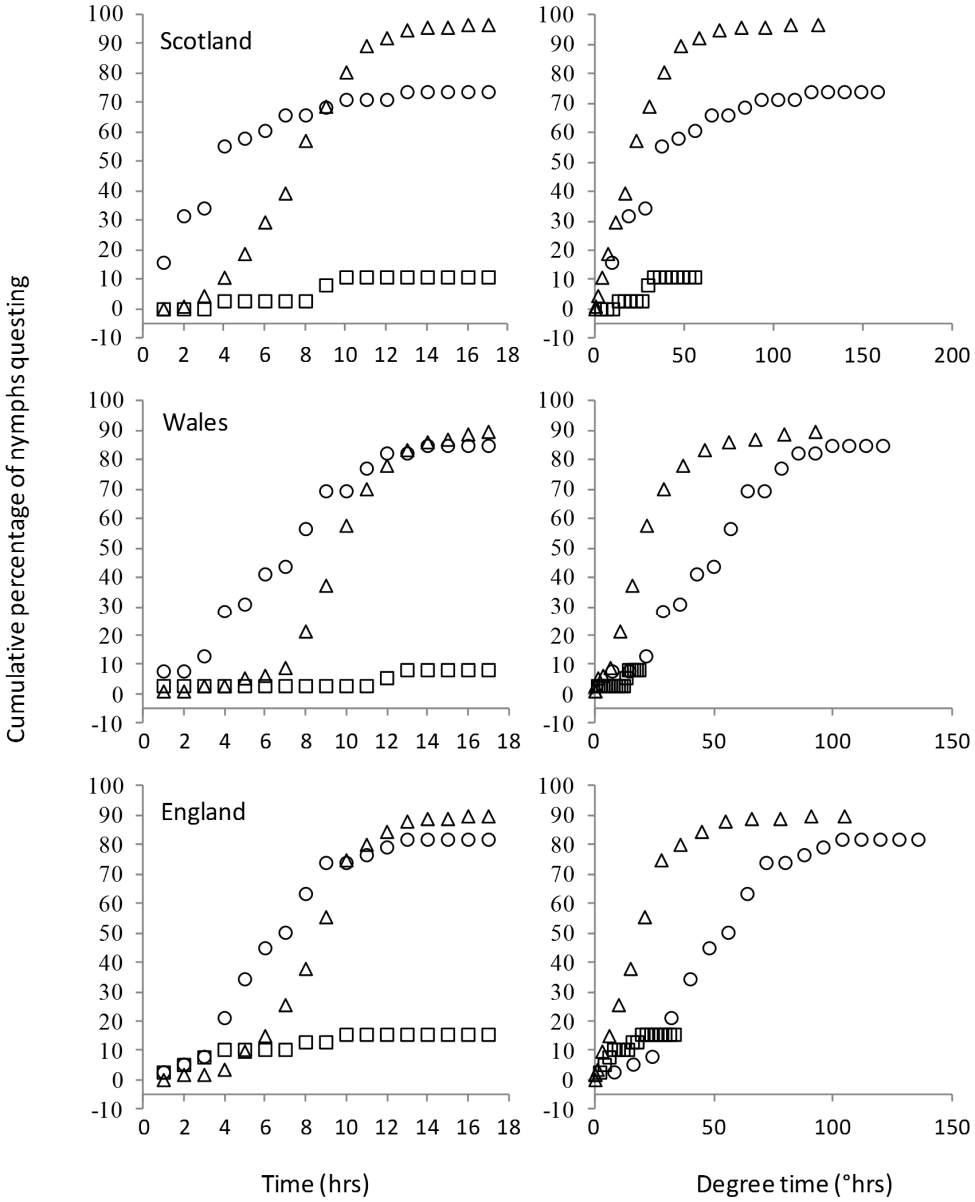
Comparison of the three UK populations in which temperature was increased hourly (Δ) by 1°C from 5°C or held constant at 15°C (○) or constant at 9°C (□) for 17 hours. In the left-hand panel cumulative questing is shown against time; in the increasing temperature experiment 9°C and 15°C were at 5 and 11 hours respectively. In the right-hand panel cumulative questing is shown against degree-time.

The average time taken for ticks to respond to a constant temperature of 15°C was significantly shorter for ticks from Scotland (3.9±0.6 hrs) compared to the ticks from Wales (6.8±0.5 hrs) and England (6.5±0.5 hrs; GLM, F_2,89_ = 7.658, P<0.001; Tukey test Wales vs England P = 0.9). We did not analyse the shapes of the response curves to a constant temperature because the Scottish population showed no lag phase and started responding within the first hour, while the other populations showed a lag phase. Under these conditions a statistical description of the curves is difficult and we rely on the GLM above and Figure 4. The sample size for responding individuals at 9°C was too small to warrant formal analysis (4, 3 and 7 responding ticks for Scotland, Wales and England respectively).

We predicted that, following conversion to degree time, the cumulative questing curves of the constant and increasing temperature experiments should overlie one another if the degree time formula (see Methods) applied to the questing activity of the ticks. However, Figure 4 shows that temperature is much more influential than time since the constant temperature treatments accumulated degree-time faster than the increasing temperature treatments, but the increase in percent questing showed the reverse with much more rapid increases in the increasing temperature treatment. Furthermore, by varying *T_metab_* we were still unable to find a value that yielded overlying data. Hence the degree time concept does not adequately explain the interaction of time and temperature in the initiation of questing.

## Discussion

Here we have tested the hypothesis that the questing behaviour of *I. ricinus* ticks is an environmentally cued threshold trait and therefore that the environmental threshold model is helpful for understanding the evolution and ecology of host searching in ticks. This hypothesis has important implications for understanding the changes in *I. ricinus* host searching behaviour, how it might alter under climate change, and the implications for tick-borne disease risk.

Our protocol is similar to Vail and Smith [46] for assaying the questing behaviour of individual ticks and presents a time- and effort-effective controlled, way of investigating within-population variation in minimum temperatures and patterns of temperature dependence in questing. To date, similar data have been collected only by correlating environmental conditions with catches of nymphs on blanket drags [36]. Such methods are labour intensive and the data tend to be highly variable. Here, with relatively few ticks, we have characterized the shape of the temperature dependence of questing across populations from a range of climates. Our data complement those of Gilbert et al. [26] who found that a higher proportion of ticks quested at cool temperatures if they were from cooler climates, compared to those from warmer climates. In the latter study, populations of ticks were held in a simulation of natural conditions with a mat layer of sand and moss; questing ticks were counted as the proportion of the population that quested in response to daily increases in temperature. Hence both the constant and hourly increasing temperatures reported here, and the daily increasing temperatures reported by Gilbert et al. [26] show similar patterns in relation to the climate of origin and suggest that ticks can be brought into the lab to generate replicable assays of the variation in questing behaviour between populations.

### Acclimation vs. local adaptation

Rearing field-collected ticks in laboratory settings limits the ability to distinguish acclimation from local adaptation as the cause of differences between populations in questing [47]–[49]. For example, it may be that all ticks, regardless of their origin, have the ability to quest at the same low temperatures, but that the thermal environment that the tick experienced during development affects the temperature threshold at which they quest. Nevertheless, we would expect there to be genetic variation in both sensitivity to acclimation temperature and to ambient temperature. Therefore, our results show that, either through selection on acclimation, or on the threshold for questing, local climate and questing temperature covary between geographic populations. This is a finding that should enable more accurate modeling of the distributional shifts of ticks and tick-borne diseases with climate change.

The environmental threshold model allows for the possibility that additional cues might also affect questing behaviour [50]. For example, environmental factors influencing questing that have been documented in other studies include size [20], infection with *Borrelia* and physical condition, such as fat stores [51], [52], and the time since, and the size of, the last blood meal [15]. Unless ticks can be reared under controlled conditions for at least two generations it is impossible to separate genetic from all possible environmental factors that might influence questing. Given the difficulty of captive rearing *I. ricinus* across generations, detailed quantitative genetic estimates may come most readily from molecular-genetic inference [53], [54].

### Local adaptation of questing switchpoints

Under the hypothesis that temperature switch points are heritable, and hence evolvable, the prediction is that local adaptation explains the relationship between climate and temperature switch point. The basis of this prediction is that the evolutionarily stable mean switch point is a population specific parameter determined by the fitness functions of adopting either of the phenotypic tactics (questing or not). We predicted that ticks from populations with cooler climates would initiate questing at lower temperatures than those from warmer climates and that this reflects differences in the (unmeasured) fitness functions (Figure 1). This prediction was upheld both when the average temperature at questing and when the lag phase parameter λ were examined. One exception to this was the relationship between S. England and N. Wales – initiation of questing occurred in the S. England population at a significantly lower temperature than in N. Wales even though S England has a slightly warmer climate than N Wales. It may be that this is evidence for population variation in sensitivity to the rate of temperature change, the North Wales population showing a shorter lag in their response to increasing temperature. This fits with the seasonal profile of temperatures above *T_metab_*, which in the Welsh population occurs over fewer months of the year than the English population, but results in a higher overall average temperature above *T_metab_*. Hence the characteristics of the Welsh population may allow ticks to rapidly exploit less frequent but on average warmer temperatures relative to the English population.

Under the environmental threshold model, the evolutionarily stable average switch point for questing is expected to be influenced by a number of factors, among them, the costs and benefits of questing or not at any particular temperature (manifest as temperature sensitive fitness functions Figure 1). Assuming the variation in thresholds is adaptive, this study suggests that these costs and benefits vary between populations of ticks. For example, tick populations in forests of different altitude in France clearly differ in their questing switch points, even though these forests are separated by only 50 km, suggesting that the functions relating fitness to temperature differ even over distances where genetic differences at neutral markers likely do not [55]. Replication of high and low altitude populations along with genetic analysis would be a worthy goal.

### Distribution of switch points

The normally distributed variation in temperature at initiation of questing is what we expect if the heritable variation that underlies sensitivity to the environmental cue is polygenic [27], [29]. One exception to the normal distribution of switch points was the Welsh population, which was significantly leptokurtic (i.e. data are more concentrated around the mean than expected [56]). Leptokurtosis may occur if the switch point distribution is ‘pressed’ up against the minimum temperature for activity e.g. [34]. Indeed theoretically there is no reason why switch points below *T_metab_* cannot occur, the result being that when *T_metab_* is reached a large proportion of the population immediately responds by questing. Whether this leptokurtosis is a replicable characteristic of this population is open to question; but it does fit, with the pattern of infrequent but warm weather seen in this population compared to the more consistent mild winter temperatures of southern England.

We found that the high altitude population from France harboured more variation in questing than the other populations. This might be expected for two reasons. First, the ticks at this site are likely to experience more variance in the temperatures above *T_metab_*, with overnight and winter temperatures dropping further than in lowland areas yet solar radiation still producing warm microclimates in the summer months. Second, increased variance could arise from gene flow from surrounding lowland areas compromising the adaptation of the population to local conditions [57]. The low levels of genetic structure in *I. ricinus* in mainland Europe [55] and the ease with which ticks can be transported over considerable distances by hosts such as deer suggest that a pattern of retarded adaptation to local environments may occur where the landscape has large altitudinal variation over short distances [57].

### The degree time hypothesis

At a constant temperature of 15°C Scottish ticks took less time before they started questing than did the Welsh and English populations. Assuming an evolved difference, this suggests that Scottish ticks have been selected for rapid responses to warm temperatures. This is likely to be favored when the opportunities for questing are fleeting, as would be the case in these more northern populations. Similar reasoning might explain why the French populations reached lower asymptotes with increasing temperature treatments – because they had not experienced enough time at warm temperatures to respond, since warm temperatures are very common in summer in those more southern areas. In general, the differences between populations in the time taken to respond to the same temperatures are concordant with the differences between populations seen in the hourly increasing temperature trial.

At 15°C many nymphs took well over an hour to initiate questing (Figure 4). This suggests that there is a general effect of time as well as temperature, where temperature has to accumulate over time in order for questing to be initiated. Hence we hypothesized that the temperature dependence of questing of *I. ricinus* might reflect the integration of time, in a similar manner to development in other Arthropods [32]. We predicted that if the switch point distribution reflected the integration of time and temperature according to the degree time concept, that the results from the constant and increasing temperature treatments would overlie one another when the cumulative frequency of questing ticks was plotted as a function of degree time. Instead the constant temperature treatment curves fell to the right of the increasing temperature curves (Figure 4), indicating that the formula accumulated degree time too fast in constant temperature conditions. Furthermore, the constant low temperature treatment the proportion of ticks questing became asymptotic at a low value, rather than gradually increasing as would be expected if time above *T_metab_* was a significant driver of questing. Finally, the increasing temperature treatments achieve higher asymptotes than constant 15°C treatment despite having less degree time overall (figure 4). Hence questing in *I. ricinus* is based largely on current temperature, making predictions about temperature and questing easier. Nevertheless, if there was no time component at all we would expect the proportion of ticks questing to always reflect the proportion of the switch point distribution that was below the temperature experienced. However, in both the high and low temperature treatments questing continued to increase over time before reaching the asymptote. Hence time above a critical temperature does have some role but it is subtle and requires further investigation to understand its effects – which may vary with climate.

## Conclusions

Our cross population study reveals how the individual behaviours of ticks can be compared in controlled conditions, to give insights into climatic variation, opening the door to detailed studies of the factors affecting the physiology, ecology and evolution of tick activity. The Richards equation provides an informative method of comparing the response of ticks to temperature, with the λ parameter in particular indicating the temperature at which a sudden increase in questing occurs. From our study, several lines of enquiry seem fruitful. Foremost among these is that an estimate of the heritability of the temperature sensitivity of questing becomes a possibility, for example by combining assays like ours with variation at neutral genetic markers. Such a study would provide insights into the potential and rate at which populations might adapt to changes in climate. Our results suggest that the environmental threshold model can provide a useful framework for future work on the evolutionary ecology of tick behaviour and the transmission of tick-borne diseases as climate changes.
